# The role of polyamine metabolism in remodeling immune responses and blocking therapy within the tumor immune microenvironment

**DOI:** 10.3389/fimmu.2022.912279

**Published:** 2022-09-02

**Authors:** Jiachun Lian, Yanfang Liang, Hailiang Zhang, Minsheng Lan, Ziyu Ye, Bihua Lin, Xianxiu Qiu, Jincheng Zeng

**Affiliations:** ^1^ Guangdong Provincial Key Laboratory of Medical Molecular Diagnostics, The First Dongguan Affiliated Hospital, Guangdong Medical University, Dongguan, China; ^2^ Institute of Laboratory Medicine, School of Medical Technology, Guangdong Medical University, Dongguan, China; ^3^ Department of Pathology, Dongguan Hospital Affiliated to Jinan University, Binhaiwan Central Hospital of Dongguan, Dongguan, China; ^4^ Dongguan Metabolite Analysis Engineering Technology Center of Cells for Medical Use, Guangdong Xinghai Institute of Cell, Dongguan, China; ^5^ Key Laboratory of Medical Bioactive Molecular Research for Department of Education of Guangdong Province, Collaborative Innovation Center for Antitumor Active Substance Research and Development, Zhanjiang, China; ^6^ Department of Biochemistry and Molecular Biology, School of Basic Medicine, Guangdong Medical University, Zhanjiang, China

**Keywords:** polyamine, tumor immune microenvironment, metabolism, T cell, immunotherapy, innate immune, adaptive immune

## Abstract

The study of metabolism provides important information for understanding the biological basis of cancer cells and the defects of cancer treatment. Disorders of polyamine metabolism is a common metabolic change in cancer. With the deepening of understanding of polyamine metabolism, including molecular functions and changes in cancer, polyamine metabolism as a new anti-cancer strategy has become the focus of attention. There are many kinds of polyamine biosynthesis inhibitors and transport inhibitors, but not many drugs have been put into clinical application. Recent evidence shows that polyamine metabolism plays essential roles in remodeling the tumor immune microenvironment (TIME), particularly treatment of DFMO, an inhibitor of ODC, alters the immune cell population in the tumor microenvironment. Tumor immunosuppression is a major problem in cancer treatment. More and more studies have shown that the immunosuppressive effect of polyamines can help cancer cells to evade immune surveillance and promote tumor development and progression. Therefore, targeting polyamine metabolic pathways is expected to become a new avenue for immunotherapy for cancer.

## 1 Introduction

**Figure 1:**
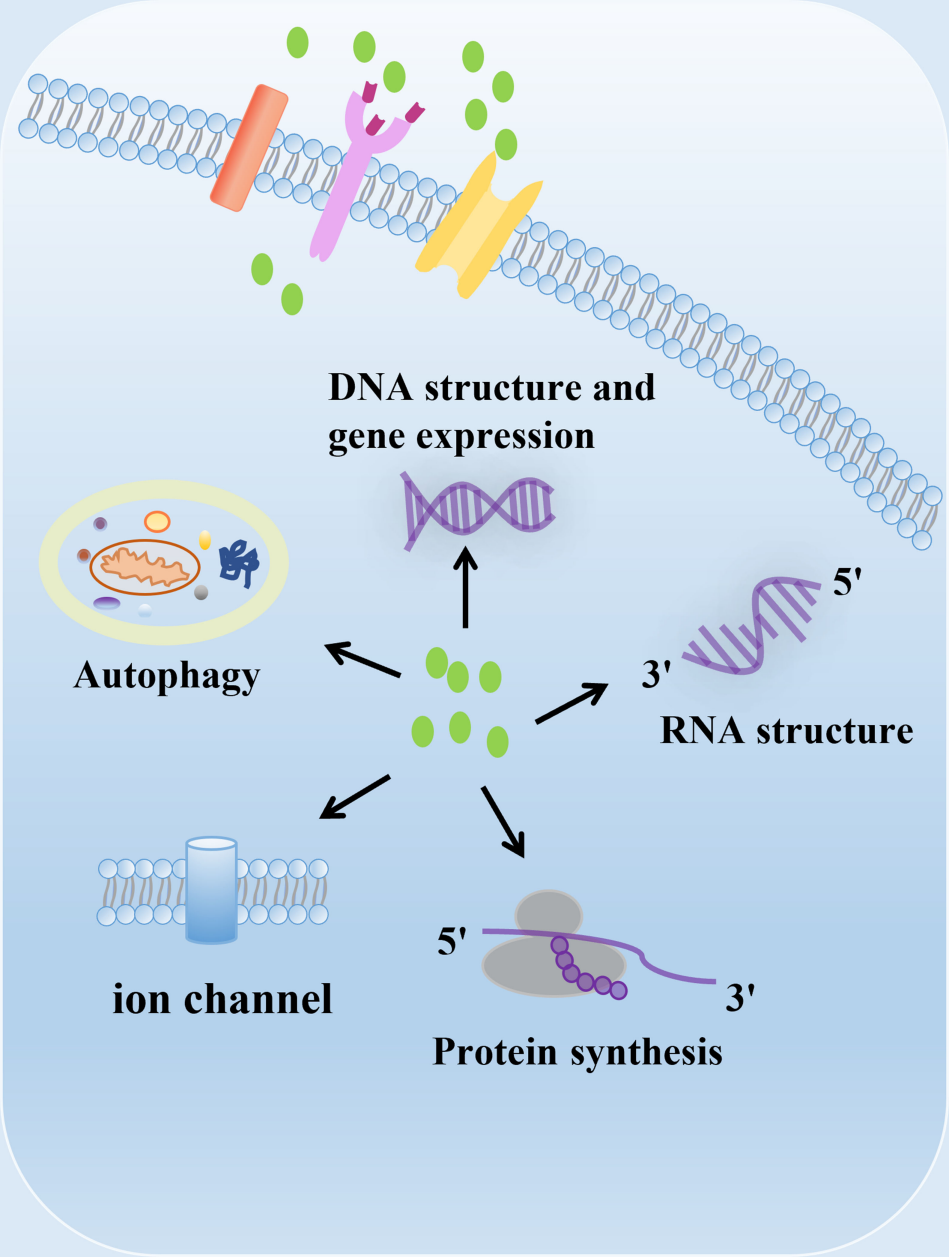
The biological function of polyamines. Polyamines have multiple roles in cells, including regulation of gene expression, RNA structure, protein synthesis, ion channel flux, and autophagy. Polyamines are required for growth and play important roles in a variety of physiological processes, including immunity, aging, hair growth, and wound healing.

Polyamines, including putrescine, spermidine and spermine, are polycationic alkylamine that present in mammalian cells in millimolar concentrations (1). They can interact with negatively charged biological macromolecules such as nucleic acids and neurotransmitter under physiological pH conditions (1) (**Figure 1**). Polyamines are reported to be involved in regulation of DNA synthesis and stability, transcription, ion channel transport, and protein phosphorylation (2–5). In mammals, polyamines play important roles in diverse physiological processes, including immunity, aging, hair growth, and wound healing (1). The intracellular concentration of polyamines varies greatly depending on cell types, cellular context and the surrounding microenvironment (6, 7). Polyamines are necessary for normal cell growth, and their consumption results in cell stasis. In the early stages of tumor transformation and progression, multiple carcinogenic pathways lead to the dysregulation of polyamine demand and metabolism, indicating that elevated levels of polyamines are necessary for transformation and tumor progression (8, 9).

Human diet and gut microbiota are also important sources of polyamines (10–12). Polyamines are present in all types of foods in a wide range of concentrations (13). The predominant polyamine in plant-derived foods is spermidine, whereas animal-derived foods have higher levels of spermine (13). Studies have shown that dietary polyamines intake is associated with cardiovascular, intestinal development, cancer progression, and anticancer immunity (14, 15). Oral supplementation of spermidine in mice can prolong life span, enhance cardiac autophagy, and improves the mechanical elastic properties of cardiomyocytes (16). Exogenous spermidine supplementation also reduces transplantable tumor growth, stimulates anticancer immune surveillance in combination with chemotherapy, and inhibits tumorigenesis in mice caused by chemical injury (17). Furthermore, Carlos Gómez-Gallego et al. reported that formula-fed mice supplemented with polyamines were similar to normal breast-fed mice in terms of microbial communities, lymphocyte numbers, and immune-related gene expression throughout the gastrointestinal tract (18). Gut microbial-derived polyamines are another important source of host polyamine reservoirs. Gut microbes can synthesize putrescine, spermine, and spermidine in milligram concentrations and use polyamines for cell-to-cell communication, cell signaling, and cell differentiation (19). Bacteria colonizing the gut produce polyamines, primarily through the transamination of ingested amino acids by catalytic enzymes, especially arginine (20, 21). Studies have shown that supplementation with arginine and/or *Bifidobacterium animalis subsp. lactis* LKM512 increases the content of polyamines in the intestine of mice and significantly prolongs lifespan, which is related to the down-regulation of inflammation-related genes and the improvement of intestinal barrier function (22, 23). With the in-depth study of polyamines derived from gut microbes, the presence of probiotics was found to increase the concentration of polyamines in the gut (24). Studies have shown that consuming yogurt containing the probiotic strain *B. animalis subsp. Lactis* LKM512 can increase the concentration of polyamines in human intestine, which is beneficial to improve intestinal health, prolong life and quality of life (25–27). Moreover, consumption of LKM512 yogurt can improve the intestinal environment and induce T-helper type 1 cytokine (IFN-gamma) in atopic dermatitis (AD) patients (25), which also suggests the potential role of probiotic-derived polyamines in immune regulation.

Tumors are complicated multicellular systems characterized by the sophisticated interaction between cancer cells and the tumor microenvironment (TME) (28). TME consists of extracellular matrix (ECM) and various noncancerous cell types, including immune cells, endothelial cells, pericytes, and fibroblasts (29). In tumor immune microenvironment (TIME), including various T helper cells, monocytes/macrophages, natural killer (NK) cells, neutrophils, and dendritic cells, have multifaceted roles during carcinogenesis and progression (30). TME, characterized by either elevated and chronic inflammation or immunosuppression, is considered as one of the hallmarks of cancer (31). In order to survive and proliferate in TIME, tumor cells need to evade immune surveillance and avoid being killed by cytotoxic lymphocytes. This is achieved by shaping the TIME into a tolerable and immunosuppressive environment, which is characterized by impaired production of tumoricidal cytokines and chemokines, decreased infiltration of activated T lymphocytes, cytotoxic CD8^+^T cells, and NK cells, and increased infiltration of immature myeloid derived suppressor cells (MDSC), regulatory T cells (Tregs), and other immunosuppressive cells (32–36).

Increased polyamine metabolism is commonly observed in various types of cancer. Elevated levels of polyamines stimulate cell proliferation and angiogenesis in tumors, thereby promoting tumorigenesis and development (37–40). Multiple oncogenes and tumor suppressors regulate tumor polyamine metabolism, which not only increased polyamine biosynthesis but also increased cellular uptake of polyamines *via* an upregulated polyamine transport system (41, 42). To date, many reports have suggested that polyamines play a functional role in immune-modulation, and participate in anti-tumor immune response by regulating the proliferation, differentiation and function of immune cells. Polyamines are essential for the activation and proliferation of mouse CD4^+^ and CD8^+^ T lymphocytes (43). In mouse bone marrow derived macrophages, spermidine-dependent OXPHOS metabolism may be beneficial to the alternative activation of ARG1 expression and inhibition of pro-inflammatory cytokine expression, which reduces the infiltration of autoimmune CD4^+^ and CD8^+^ T lymphocytes into the central nervous system and the clinical score of experimental autoimmune encephalomyelitis (44, 45). Polyamines can also improve anti-cancer immunity through autophagy, a cellular metabolic process necessary for T cell activation, function and survival (46–50). However, polyamines have also been reported to exert immunosuppressive effects, which may contribute to the multiple complex mechanisms by which cancer cells escape from immune responses. Myeloid-derived suppressor cells (MDSC) in the tumor microenvironment utilize polyamines to invoke their suppressive activations and support their metabolism (51–56). Polyamines also inhibit lymphocyte proliferation, reduce neutrophil locomotion and NK cell activity, and suppress macrophage-mediated tumoricidal activity through reprogramming proinflammatory M1 to anti-inflammatory M2 phenotypes (57–61). Taken together, polyamine metabolism and its metabolic molecules, play a complex role in the differentiation and function of various immune cells under both physiological and pathological conditions.

Metabolic regulation is a key component of coordinating the immune response (62). Targeting polyamine metabolism has long been an attractive approach for cancer chemotherapy. In animal experiments, polyamine deprivation enhances the production of chemokines, reverses the inhibitory activity of cytotoxic cells induced by tumor inoculation, and prevent tumor-induced immunosuppression (59, 63). Some studies have shown that inhibition of ornithine decarboxylase (ODC), and/or treatment of polyamine transport inhibitors (PTIs), significantly reduces the tumor growth rate due to the enhanced anti-tumor immunity (64–66). Moreover, polyamine blocking therapy (PBT) reduces polyamine-mediated immunosuppression in the tumor microenvironment and activates tumor-killing T cells (67). Since accumulating evidence supports that polyamines contribute important roles to immune evasion of tumor cells, polyamines might be added to the list of immunosuppressive metabolites (68). In this review, we outline the relationship between polyamines and immune cell function. We also discuss the impact of polyamines on the tumor immune microenvironment, and the dual regulatory functions of polyamines in cancer and immune cells. Finally, we provide insights on targeting polyamine metabolism as a novel avenue for cancer immunotherapy.

## 2 Polyamine metabolism

Under normal physiological conditions, the intracellular concentration of polyamines is strictly regulated by biosynthesis, catabolism and transport mechanisms (7, 69, 70). While polyamine pathways, which are modulated by several important oncogenic pathways, are often dysregulated in cancer. As such, polyamine metabolism may serve as a promising target for anticancer therapies (9).

### 2.1 Polyamine biosynthesis

**Figure 2:**
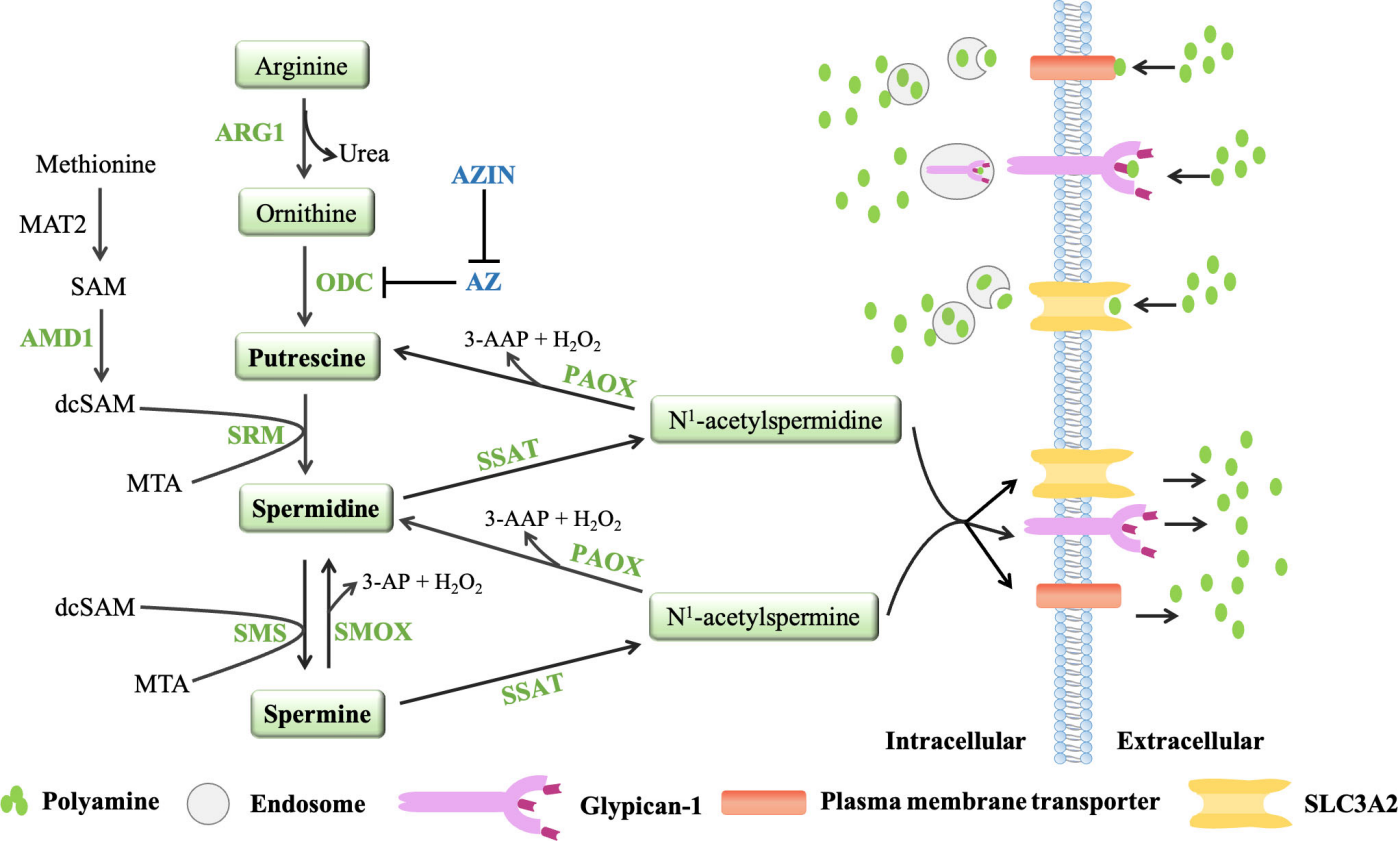
The polyamine metabolic pathway and transport way. Polyamine biosynthesis involves the conversion of ornithine to putrescine by ornithine decarboxylase (ODC), followed by the formation of spermidine *via* spermidine synthase (SRM) and decarboxylated s-adenosylmethionine (dcSAM, formed by AMD1). The aminopropyl fragment required for putrescine to produce spermidine was provided by dcSAM. In a similar manner, spermine is produced from the conversion of spermidine by spermine synthase (SMS) and AMD1. The polyamine catabolism process occurs through the action of amine oxidase, mainly polyamine oxidase (PAOX) and spermine oxidase (SMOX). PAOX and SMOX can generate a large amount of reactive oxygen species (ROS) during the process of decomposing polyamines, causing oxidative damage. Currently, three models of polyamine transport systems have been proposed. Although the molecules involved in the polyamine transport system have not been fully recognized, it is known that the polyamine transport system is energy dependent and substrate selective. ODC antienzymes (AZs) and antizyme inhibitors (AZINs) also play important roles in polyamine transport. ODC monomers have a higher affinity for AZs. When the intracellular polyamine concentration is high, AZs binds to ODC monomers, preventing ODC activity and promoting the binding of ODC monomers to the 26S proteasome for degradation in a ubiquitin (Ub)-independent manner (only AZ1 induces ODC degradation). However, the binding of AZs to ODC can be blocked by AZINs.

Polyamines are produced from arginine and ornithine, which are controlled by *de novo* synthesis and diet (71, 72) (**Figure 2**). Ornithine is produced from arginine by arginase 1(ARG1) and metabolized by ornithine decarboxylase (ODC) to produce putrescine, which is the first mammalian polyamine (73). Methionine is metabolized by methionine adenosyltransferase (MAT2) to produce s-adenosylmethionine (SAM), which is the main methyl donor for cell methylation (74). SAM is decarboxylated by adenosylmethionine decarboxylase 1 (AMD1) to produce decarboxylated SAM (dcSAM), which is a substrate for polyamine synthesis (72). In inflammatory and autoimmune diseases, intracellular methylation modification affects immune dysfunction in the body, including CD4^+^T lymphocytes, CD8^+^T lymphocytes, B lymphocytes, macrophages, and regulatory T cells (75). Therefore, in addition to playing an important role in the synthesis of polyamines, AMD1 may also affect the methylation reaction by affecting the availability of SAM, and even play a role in immune function (75). Decarboxylated SAM (dcSAM) is the aminopropyl donor, which is added to the reactions catalyzed by spermidine synthase (SPDSY, coded by SRM) and spermine synthase (SPMSY, coded by SMS) to convert putrescine into polyamine metabolites (73, 76). Spermidine synthase (SRM) catalyzes putrescine and dcSAM to produce spermine and methylthioadenosine (MTA). Spermidine can react with the second dcSAM molecule through the action of spermine synthase (SMS) to produce spermine and another MTA molecule (69).

### 2.2 Polyamine catabolism

Polyamine catabolism is another key factor in maintaining polyamine homeostasis (**Figure 2**). The aminopropyltransferase reaction to form spermidine and spermine is irreversible, but the interconversion of polyamines in cells can occur through the action of amine oxidase, which are mainly polyamine oxidase (PAOX) and spermine oxidase (SMOX) (77). The activity of PAOX is limited by the availability of acetylation products produced by spermidine/spermine N1-acetyltransferase 1 (SSAT, which is encoded by SAT1). SSAT is a highly inducible enzyme, which is regulated in response to the free polyamine concentration to maintain polyamine homeostasis (78). SSAT forms N1-acetylspermine and N1-acetylspermidine by adding acetyl group to the N1 position of spermine or spermidine from acetyl-coenzyme A. Depending on the initial substrate, these acetylated polyamines can be excreted from the cell or converted to 3-acetylaminopropanal, H_2_O_2_ and spermidine or putrescine by PAOX (78). SMOX is an FAD-dependent enzyme with high homology to PAOX and exists in the cytoplasm and nucleus. Unlike PAOX, SMOX directly oxidizes spermine to generate 3-aminopropanal, H_2_O_2_ and spermidine (77). These catabolic pathways can prevent excessive concentrations of polyamines in cells. PAOX and SMOX can generate a large amount of reactive oxygen species (ROS) during the process of decomposing polyamines, causing oxidative damage (77, 79).

### 2.3 Polyamine transport

In addition to polyamine synthesis and catabolism, polyamine transport also plays an important role in maintaining an appropriate level of intracellular polyamines. Completely protonated at physiological pH, polyamines do not passively diffuse across cell membranes. Currently, three models of polyamine transport systems have been proposed (80) (**Figure 2**). One proposed model relies on a highly selective membrane permease to allow polyamines to be rapidly internalized into endosomes, where they can be dispersed throughout the cell as needed (81). In a second model, polyamines are internalized by endocytosis which bound to heparin sulfate moieties in glypican-1 at the cell surface. Polyamines are internalized into the endosomes and then released through an oxidation mechanism mediated by nitric oxide (82). The third model proposes that polyamine transport is mediated by endocytosis and solute carrier transport mechanisms in the gastrointestinal tract, especially SLC3A2 (82).

Ornithine decarboxylase (ODC) and ODC antizymes (AZs) also play an important role in polyamine transport (83, 84). ODC is active as a homodimer, but the ODC monomer has a higher affinity for AZ. There are three main members of the antizyme family: AZ1, AZ2 and AZ3 (85). Studies have shown that AZ2 is expressed at much lower levels compared to AZ1. However, AZ2 shows higher evolutionary conservation, which may indicate increased functional value (86, 87). AZ3 is tissue-specific and is mainly expressed in the testis during certain stages of spermatogenesis (88, 89). Moreover, AZ1, AZ2, AZ3 are able to inhibit ODC activity and polyamine uptake, only AZ1 induces ODC degradation (90). AZs negatively regulate the uptake activity of polyamines. When the intracellular polyamine concentrations are high, polyamines transport will be blocked because AZs can bind to ODC monomers to inhibit ODC activity and chaperon the ODC monomers to the 26S proteasome for degradation in a non-ubiquitin (Ub) manner. When the intracellular polyamine concentrations are low, the full-length AZ cannot be translated, so it cannot inhibit ODC activity or block the transport of polyamines (83, 84). AZ can also bind to and inhibit polyamine-specific transporters on the plasma membrane to affect the transport of polyamines (91). AZs and polyamine synthesis were also affected by the antizyme inhibitors (AZINs), which are proteins highly homologous to ODC (90), and retain no ornithine-decarboxylating activity (92, 93). In contrast to ODC, AZINs are degraded by the proteasome through a ubiquitin-dependent mechanism (94). Two subtypes of antizyme inhibitors, AZIN1 and AZIN2, have been reported. AZIN1 is required for normal embryonic development and is associated with cell proliferation, but AZIN2 is predominantly expressed in the human brain and testis, and AZIN2 may play a role in terminal differentiation rather than cell proliferation (95). Since only transfection experiments have shown that AZIN2 affects ODC activity and polyamine uptake, and little is known about the effect of AZIN2 on polyamine levels *in vivo (* 96, 97), the AZIN described in this article refers to AZIN1. AZIN1 interacts with AZ more efficiently than ODC, counteracting the negative effects of AZ on intracellular polyamine biosynthesis (98, 99). And when AZIN1 is tightly bound to AZ, AZIN1 does not degrade as fast as ODC. Conversely, AZ binding stabilizes AZIN1 by preventing AZIN1 ubiquitination (94, 100). Notably, AZIN1 can also increase extracellular polyamine uptake, presumably by binding to and sequestering AZ, thereby preventing negative regulation of polyamine transport by AZ (96). Studies have shown that AZIN is overexpressed in a variety of malignancies (gastric cancer, lung cancer, prostate cancer, liver cancer and ovarian cancer) and has carcinogenic effects (101–104). Increased AZIN1 expression correlates with elevated polyamine levels, which promote tumor cell proliferation (100, 105). Although AZ is a tumor suppressor and its expression can prevent cell growth and tumorigenesis, AZIN1 competes with ODC to release ODC from the ODC-AZ complex based on the stronger binding ability of AZIN1 and AZ, which is conducive to the polyamine synthesis pathway and promotes cancer progression (100, 106). With the deepening of research, it is found that the cancer-promoting effect of AZIN1 can also affect the secretion of cytokines in the tumor microenvironment, such as IL-8 and TGF-β (107, 108). Studies have shown that AZIN1 can up-regulate IL-8 and promote tumor angiogenesis. IL-8 has been reported to contribute to cancer progression and metastasis through different mechanisms, including preangiogenic and cancer stem cell maintenance, but its ability to attract and regulate neutrophils and macrophages is arguably one of the most important factors (107, 109). Although there is no direct evidence that AZIN can play a role in the tumor immune microenvironment, AZIN may affect the tumor immune microenvironment by regulating the secretion of cytokines.

### 2.4 Polyamine metabolites

#### 2.4.1 Putrescine

Putrescine is the precursor of spermidine and spermine, produced from ornithine by ornithine decarboxylase (ODC) (73). Putrescine regulates DNA structure, mRNA translation and protein activity, and plays an important role in promoting cell proliferation and migration (2–5). Putrescine has been shown to promote the proliferation of colon cancer cells, even be used as a biochemical marker for malignant brain tumors (110, 111). It is worthy to note that putrescine exerts anti-inflammatory function by inhibiting IL-8 and TNF-α in a LPS-stimulated inflammation model, which may provide a survival mechanism for tumor cells to evade immune response (112). Meanwhile, putrescine derived from macrophages induces 5-FU resistance in colorectal cancer (113). In addition, putrescine can also inhibit the maturation of cytolytic T lymphocyte (CTL), which may impair anti-tumor immunity (114).

#### 2.4.2 Spermidine

Spermidine is a metabolite of putrescine converted by spermidine synthase (SRM), or an oxidized product of spermine catalyzed by SMOX. Spermidine can interact with polyanions such as nucleic acid and protein to maintain DNA genome homeostasis and regulate cellular autophagy, apoptosis, oxidative stress and so on (115). There have been many reports suggest that spermidine prolongs the life span across species in an autophagy-dependent manner, and fights cancer and age-related diseases (such as cardiovascular disease, neurodegeneration) (16, 17, 45, 116). In the tumor microenvironment, spermidine can exert multiple functions, e. g. the cell-autonomous inhibitory effect on proliferation or induction of apoptosis of cancer cells by releasing H_2_O_2_ and reactive aldehydes, impeding communication between cancer cells and immune monitoring effector cells, suppressing the function of immunosuppressive cells and promoting the polarization of M2-like tumor associated macrophages (TAMs) (117). In addition, spermidine can also increase the autophagy-dependent release of ATP to facilitate immune monitoring (117).

#### 2.4.3 Spermine

Spermine is converted from spermidine by spermine synthase. Spermine also regulates cell proliferation, differentiation, and apoptosis (7, 118). Spermine is more effective against reactive oxygen species and other stresses than spermidine and has been shown to be involved in the maturation of the body’s immune system and induction of autophagy to delay brain aging (119–121). In addition, spermine has been reported to regulate T cell function (122), and dietary supplementation of spermine reduces inflammatory response, enhances immune function, and regulates gene expression of inflammation-related signal molecules (123).

## 3 Roles of polyamines in the innate immune cell responses in TIME

### 3.1 Regulation of macrophage polarization by targeting polyamine-eIF5A-hypusine axis

Macrophage are professional phagocytic cells that internalize large particles such as debris, apoptotic cells, pathogens, and maintain a stable environment in the body (124). According to their functions, macrophages can be classified into two categories: classically activated or inflammatory M1 macrophages and alternately activated or anti-inflammatory M2 macrophages (125, 126). The cytokines released by cancer cells in the tumor immune microenvironment (TIME) affect the polarization of macrophages. In the early stages of tumor formation, M1 macrophages in TIME initiate inflammation and exert anti-tumor immunity (126). However, in established tumors, M1 macrophages can be reprogrammed into M2-like TAMs by cytokines enriched in TIME, such as IL-10, IL-4, and IL-13, etc. (125). M2 macrophages have anti-inflammatory effects and can promote angiogenesis and fibrosis, so they have immunosuppressive activity (124). The macrophages located around the TIME are often called TAMs. However, TAMs are mostly M2 macrophages, which play an important role in the establishment of immunosuppressive tumor microenvironment, metastasis, therapy-resistance, and recurrence of cancer (127–131). Therefore, macrophages represent a group of cells with high plasticity, which can constantly shift their functional states in response to subtle changes in tissue physiology or environmental challenges (132–137).

Numerous studies have implicated the involvement of polyamines in regulating polarization and functions of macrophages, particularly, in regulating tumor immunity (138). For instance, putrescine has been shown to inhibit M1 macrophage activation (112, 138) through downregulating IL-8 and TNF-α expression in a LPS-stimulated inflammation model, thus implying the contribution of M1 macrophage inhibition to immune evasion of tumor cells (138). Spermidine inhibits M1 macrophages by reducing the expression of co-stimulatory molecules (CD80 and CD86) in macrophages and the production of pro-inflammatory cytokines (45). Moreover, spermidine induces the expression of ARG1 in macrophages and promotes the polarization of macrophages to M2 phenotype through inducing mitochondrial superoxide-dependent AMPK activation, Hif-1α up-regulation and autophagy (45, 139). In addition, spermine inhibits iNOS in macrophages activated by Helicobacter pylori to prevent the antibacterial effect of NO, leading to the persistence of cellular bacteria and an increased risk of gastric cancer (140). Spermine also induces the autophagy of liver-resident macrophages (Kupffer cells) by upregulating ATG5 expression, thereby inhibiting the pro-inflammatory M1 polarization and promoting the anti-inflammatory M2 polarization of macrophages (141).

The role of key enzymes in polyamine metabolism on the polarization and the immune functions of macrophages should not be underestimated. In tuberculosis, highly expression of ARG1 in macrophages leads to collagen deposition and lung damage, which drives to inflammation by inhibiting Th1 cells (142). In colitis, ODC in macrophages exacerbates colitis and promotes the occurrence of colitis-related colon cancer by impairing the immune response of M1 macrophages (143). During the occurrence and development of human esophageal squamous cell carcinoma (ESCC), the activation of ODC can increase the secretion of IL-33 in the tumor site, thereby promoting the polarization of macrophages to the anti-inflammatory M2 phenotype (144). Moreover, MTA accumulates in MTAP-deficient tumor cells, blocks the activation of macrophages and inhibits the production of TNF-α through adenosine A2 receptor and TLR receptor after LPS stimulation, which promotes the differentiation of M2 macrophages with immunosuppressive effect (145).

According to the recent research reports, polyamines can regulate the activation and function of macrophages largely depends on the arginase-eIF5A-hypusine axis. The researchers activated mouse bone marrow-derived macrophages with IL-4 [referred to M(IL-4)], and found that eIF5A^H^ (eIF5A Hypusination) was induced upon activation with IL-4. Significantly increased eIF5A^H^ in M(IL-4) correlated with enrichment of hypusinating enzymes (ODC, DHPS, DOHH) expression in these cells. It was also observed that increased arginine in M(IL-4) promoted putrescine production by ODC and increased flux of putrescine to spermidine, which could be used to synthesize hypusine. These data may imply that even if the expression of polyamine-hypusine enzymes is not altered, hypusine synthesis might increase due to the increased availability of ornithine, putrescine and spermidine, followed by changes in eIF5A^H^ levels in immune cells (44). In conclusion, various links in the polyamine pathway play important roles in the immunomodulatory function of macrophages, especially the activation of macrophages, thereby promoting the establishment of an immunosuppressive tumor microenvironment.

### 3.2 Excessive polyamines in cancer cells confer immunosuppressive properties on DCs

Dendritic cells(DCs) are bone marrow-derived cells that present in all tissues (146–148), and are sentinels of the immune system, which play a central role in linking innate and adaptive immune responses (146). The function of DCs is determined by the integration of environmental signals, which are sensed *via* the surface expression and intracellular receptors of cytokines, pathogen-associated molecular patterns (PAMPs) and damage-associated molecular patterns (DAMPs) (149). Dendritic cells can capture tumor antigens released from live or dead tumor cells, and cross-present these antigens to T cells in the tumor draining lymph nodes, thus leading to the generation of tumor-specific CTLs (150, 151). However, signals from the TIME can prevent antigen presentation and the establishment of tumor-specific immune responses *via* a variety of mechanisms. For example, the anti-inflammatory cytokine IL-10 secreted by immunosuppressive cells can inhibit the maturation of DCs, leading to antigen-specific anergy (152, 153). In addition, the tumor antigens, e. g. glycoproteins carcinoembryonic antigen (CEA) and mucin 1(MUC1), can be endocytosed by DCs and confined to the early endosomes, thus preventing their effective processing and presentation to T cells (154). Polyamines also play an important role in the maturation and functional regulation of DCs. ARG1, a key enzyme of polyamine biosynthesis, is highly expressed in DCs, and is one of the most important immune checkpoints that allow tumor immune escape (155–158). It has been reported that DCs metabolize local arginine to produce local arginine starvation and prevent the progression of T cell cycle in the G0-G1 phase by impairing the expression of the T cell receptor (TCR) CD3-ξ chain in human and mouse cells (159, 160). In the psoriatic inflammatory circuit, lack of Pp6 in keratinocytes causes ARG1 accumulation and drives polyamine production, which promotes self-RNA sensing by dendritic cells, leading to increased inflammation (161). Adding putrescine to the microenvironment of DCs will hinder their ability to effectively cross-prime exogenous antigens, indicating that their immunogenic functions are reduced (162). Spermidine activates the Src kinase and confers IDO1-dependent immunosuppressive properties in DCs (163). Moreover, spermine and spermidine may convert immunogenic DCs into tolerant DCs by promoting the production of IL-10, thereby inducing anergic cytotoxic CD8^+^T cells (164–166). Spermidine may also inhibit the differentiation and maturation of DCs by promoting the production of VEGF (167–169). In addition, ROS is released during polyamine catabolism (77, 79). High levels of ROS in the tumor microenvironment may inhibit the function of DCs. ROS can enter DCs through diffusion across the plasma membrane or extracellular vesicles released by tumor cells, which gives the tumor microenvironment more opportunities to inhibit DC function (170). Therefore, the ROS generated during the catabolism of polyamines may not only inhibit the cross-presentation of DCs, but also inhibit the maturation of DCs through endoplasmic reticulum stress (171, 172).

### 3.3 Polyamines for NK cells: A double-edged sword

NK cells are the first subtype of innate lymphoid cells (ILCs) characterized by a surface marker profile CD3^−^CD56^+^NKp46^+^ in humans, exerting natural cytotoxicity against primary tumor cells and metastasis by inhibiting proliferation, migration and colonization to distant tissues (173). The detection of abnormal cells by NK cells is determined by the integration of complex signals such as IL-12, IL-15, and IL-18, as well as the balance between activation and inhibition signals and the interaction of MHC-I on the surface of target cells (174–176). During infection and inflammation, NK cells are recruited and activated within a short period of time, proliferate rapidly and largely contribute to the innate and adaptive immune response (177, 178). NK cell activation is inhibited by the binding of inhibitory receptors to class I HLA (MHC I) molecules. However, many cancer cells downregulate the expression of the MHC I molecules to evade the detection of cytotoxic CD8^+^T cells. Therefore, due to the lack of MHC I-induced signaling *via* inhibitory receptors and the subsequent increase in activation signaling, NK cells can recognize and respond to cells of this missing-self phenotype, and ultimately lead to target cell lysis (179).

Despite their activity in controlling tumor growth, NK cells are susceptible to multiple immunosuppressive mechanisms in TIME. Many cancer-related soluble immunosuppressive molecules have negative effects on NK cell function, including TGF-β, IL-10, indoleamine 2,3-dioxygenase, prostaglandin E2 (PGE2) and macrophage migration inhibitory factor (MIF) (180). In addition to immunosuppressive cytokines, accumulation of tumor-derived metabolites in TIME, including polyamines, also exerts immunosuppressive effects on NK cells (37–40, 68, 181). Polyamines act as a double-edged sword in regulating NK cell functions. According to reports, polyamines act as natural immunosuppressive agents by reducing the cytolytic properties of NK cells, which protect tumors from the host’s immune response (182), while polyamine deprivation stimulates NK cell activity (59). Polyamines can also inhibit the expression of NK1.1 receptors of NK cells and the production of perforin and IFN-γ, thus attenuating NK cell-mediated tumor cell recognition and cytolysis, and such effects could be reversed by treatment with DFMO, rosuvastatin, and their combination (182). Adhesion molecules have been shown to promote NK cell activation (183). Lymphocyte function-associated antigen 1 (LFA-1) is expressed on NK cells and interacts with intercellular adhesion molecules (ICAM) on target cells. The combination of LFA-1 and ICAM-1 can enhance NK cell-mediated cytotoxicity by enhancing the polarization of the cytoskeleton mechanism, which is necessary for effective delivery of cytotoxic particles (183). However, spermine, a natural polyamine, can negatively affect the expression of LFA-1 and attenuate the binding of LFA-1 and ICAM-1, thus resulting in a decrease in NK cell-mediated cytotoxicity and ineffective delivery of cytotoxic particles (183, 184). On the other hand, polyamines may participate in the differentiation of NK cells, contribute to their maturation and protect their viability. It is well known that IL-2 can induce the proliferation of NK cells and improve their cytolytic activity (185). Polyamine biosynthesis can increase IL-2 production, thus enhancing the cytotoxicity of NK cells (186). In addition, polyamines, particularly spermidine and spermine, reverse immune senescence through translational control of autophagy (121, 187). Autophagy is necessary for the differentiation of mature NK cells from bone marrow-derived HSC (188, 189), and is essential for NK cells to clear the virus and enhance the memory formation of NK cells (188–190). Therefore, polyamines are involved in regulating the differentiation process of NK cells, even play an important role in tumor immunity.

### 3.4 Polyamines, activators of type I NKT cells

NKT cells, subtypes of innate-like T lymphocytes, can quickly respond to antigen stimulation and produce a large amount of various cytokines and chemokines, thus serving as a key player in connecting the innate immune system and the adaptive immune system (191–194). Unlike the TCR of traditional T cells, which only recognizes one (or at most a few) epitopes, a single TCR of NKT cells can react with a large number of antigens, including self and foreign antigens. Therefore, in a T cell environment specific to an antigen, their numbers are high enough to initiate a significant immune response, although the absolute frequency of NKT cells is low (e.g., about 1% in mouse spleen) (195–197). According to the heterogeneity of TCR rearrangement, NKT cells are divided into two types, type I or type II NKT cells with different roles in tumor immunity (198). Usually, type I NKT cells promote tumor immunity, while type II NKT cells inhibit tumor immunity. Under normal conditions, an immunomodulatory axis exists between type I and type II NKT cells, wherein they have opposite polar functions and counteract each other (198).

In tumor immune surveillance, NKT cells can directly kill malignant cells. For example, both mouse and human NKT cells can directly lyse tumor cells through a perforin-dependent mechanism, and the expression of granzyme B also enhances the killing effect of NK cells (199, 200). However, polyamines can inhibit the production of perforin, making it unable to effectively lyse tumor cells (182). Polyamine blocking therapy (PBT) has been shown to increase the production of granzyme B in immune cells, thus enhancing the killing effect of NKT cells (67). It is reported that IL-12 is an effective inducer of IFN-γ (201), the main mechanism by which NKT cells act against cancer cells and induce other downstream effector cell functions (especially NK cells and CD8^+^ T cells) to produce more IFN-γ to mediate tumor lysis (202, 203). Polyamines have been shown to reduce the production of IL-12 and IFN-γ in immune cells (164, 182), thus contributing to the inhibition of the killing function of NKT cells and NKT-mediated induction and activation of NK cells, DCs cells, and other immune cells. A main factor of type II NKT cells-mediated tumor immunosuppression is the increased production of IL-13 and IL-4 cytokines, which tilt immune response mainly toward the Th2 type with pro-tumor functions (204). In immune cells, IL-4 and IL-13 can increase polyamine levels (68, 205) that may also contribute to type II NKT cell-mediated tumor immunosuppression. In addition to lipid antigens, type I NKT cells can also be activated through toll-like receptor (TLR)-mediated signaling (206). Polyamines have been reported to affect immune system function by participating in the expression of Toll like Receptors (TLRs). Therefore, polyamines may play an important role in regulating the recruitment and activation of type I NKT cells through TLRs (207).

### 3.5 Polyamine-PD-L1-γδ T cells: A novel immune checkpoint pathway

Gamma delta (γδ) T cells are a unique lymphocyte population that mediate natural immunity against various infections and play a unique role in immune monitoring and tissue homeostasis (208). Since γδ T cells can quickly identify infected and transformed cells, they are considered as the first line of defense against infection and malignancy (209). The main pathway of γδ T cell activation involves γδ TCR. γδ TCR can bind to soluble or membrane proteins, such as tetanus toxoid, bacterial protein, viral protein and heat shock protein (210–212). According to the TCRδ chain usage, human γδ T cells are generally divided into 2 main subgroups. One subgroup is Vδ1 T cells, which are abundant in thymus and mucosal epithelial tissues, produce a variety of cytokines such as TNF-α and IFN-γ and lyse infected or transformed target cells through cytotoxicity (213, 214). The other is Vδ2 T cells that are mainly distributed in peripheral blood and play a cytotoxic role in tumor immune regulation and virus infection (215).

γδ T cells regulate the immune function of body through the cell-to-cell contact or soluble factors such as cytokines (216). Numerous factors, such as IL-2, IL-15, IL-17, IL-21, TGF-β, and vitamin C, can regulate the differentiation of γδ T cells and their anti-tumor response (217–221). Besides, polyamines, as negative immune regulators, directly or indirectly affect the function of γδ T cells by regulating their secretion of cytokines and other mediators. eIF5A is a translation elongation factor that assists in the translation of specific transcripts, and spermidine is required for hypusination of eIF5A (44, 222). eIF5A is directly involved in the translation of IL-17, an inflammatory cytokine produced mainly by activated Th17 cells, while IL-17 produced by γδ T cells drives tumorigenesis and progression through several downstream effects on tumor cells, endothelial cells, and other immune cells (223–225). Therefore, spermidine may regulate the production of IL-17 in γδ T cells through eIF5A and participate in the immune regulation of a variety of cancers. Blocking intracellular polyamines with DFMO can significantly induce TGF-β mRNA expression and increase TGF-β content (226). TGF-β changes the adhesion characteristics of γδ T cells and plays an important role in promoting the migration ability and tissue homing of γδ T cells (227). Therefore, the occurrence and development of cancer is usually accompanied by an increase of polyamines, which may inhibit the toxic activity of γδ T cells. In recent years, researchers have discovered that γδ T cells can promote tumor promotion by regulating PD-1/PDL-1 (228). The immune checkpoint molecule PD-1 and its ligand PDL-1/2 are one of the main regulatory mechanisms that temper tumor immunity (229, 230). *In vitro* studies have shown that tumor-infiltrating γδ T cells inhibit αβ T cell activation *via* cell-to-cell contact by PD-1/PD-L1 (228), and polyamine blockade therapy has been reported to enhances the antitumor efficacy of PD-1 blockade (231), which indicates that polyamines may affect the immune function of γδT cells through PD-1/PD-L1, thereby inhibiting the activation of αβ T cells, and ultimately promote tumor progression.

## 4 Role of polyamines in the adaptive immune responses in TIME

Tumor infiltrated lymphocytes (TILs) play an important role in the establishment of a pro- or anti-tumorigenic TME (232). T lymphocytes are usually the major components of TILs, among which CD4^+^ T helper cells (e.g., Th1), CD4^+^CD25^+^ regulatory T cells (Tregs), CD8^+^ cytotoxic T cells are frequently observed in various cancers (233–235). Clinically, TILs can be separated, screened and amplified *in vitro*, and then implanted into the patient’s body to exert a specific killing effect on the tumor (236).

### 4.1 Polyamine for CD8+ tumor-infiltrating lymphocytes: TIME’s “enemy”

CD8^+^ tumor-infiltrating lymphocytes play a key role in the host’s anti-tumor immune response by acting as cytotoxic cells through the release of granzyme B, perforin, and pro-inflammatory cytokines such as TNF-α, IFN-γ, and IL-12 (237, 238). However, many factors, such as indoleamine-2, 3-dioxygenase (IDO), PD-L1, cytokine milieu, and the state of protein kinases in TIME, can suppress the infiltration and cytotoxic activities of CD8^+^ T cells and eventually lead to immune evasion by tumor cells (239–241).

T lymphocytes obtain energy for their survival, proliferation, and biological functions through various metabolic pathways, while dysregulated metabolism in TME contributes to aberrant functions of TILs, including CD8^+^ cytotoxic T cells (242, 243). Alterations in different metabolic pathways in TME can lead to exhaustion, impaired effector functions and survival of CD8^+^ cytotoxic T cells in various types of cancer (244–246). Previous studies have indicated that increased polyamine metabolism is also involved in regulation of the survival and effector function of CD8^+^ TILs (68, 247). For example, polyamines and polyamine oxidation products may inhibit the activation and proliferation of CD8^+^ TILs by down-regulating the production of IL-2 (248, 249). Increased polyamine production was associated not only with increased IL-10 levels, but also with decreased IL-12 levels, suggesting that polyamines may inhibit the cytotoxic function and cause deficiency of CD8^+^ TILs (250–252). In addition, polyamines can also reduce the expression of chemokines, thus inhibiting the migration and recruitment of CD8^+^ TILs, a key step for anti-tumor response (45, 253, 254). It has been reported that the expression of T cell co-inhibitory molecules (PD1, PD-L1 and CTLA-4) can induce exhaustion of effector T cells, while blockade of PD-1/PD-L1 T cell co-inhibitory axis can efficiently enhance the infiltration of CD8^+^ T cells into TIME and restore the anti-tumor immune response (255, 256). Most recently, several lines of evidence have shown that polyamine blocking therapy (PBT) can improve the anti-tumor efficacy of PD-1 blockade along with an increase in tumor infiltration of granzyme B^+^, IFN-γ^+^ CD8^+^ T-cells and a decrease in immunosuppressive tumor infiltrating cells including Gr-1^+^CD11b^+^ myeloid derived suppressor cells (MDSCs), CD4^+^CD25^+^ Tregs, and CD206^+^F4/80^+^ M2 macrophages (231, 257, 258). These findings suggest that polyamines are directly or indirectly involved in regulating the function of CD8^+^ TILs. Adenosine is a mediator of TME immunosuppression, and its physiological activity is mediated by adenosine receptors (ARs). It may limit the success of immunotherapy, especially the adoptive cell transfer of TILs (259–261). Activation of adenylate cyclase by inhibiting ARs can induce the increase of cellular cAMP levels (262). Studies have shown that cAMP-elevating agents have excellent anti-tumor activity, and when used in combination with other anti-tumor agents, cAMP-elevating agents show enhanced anti-tumor activity (263, 264). Furthermore, ARs inhibitors have been shown to prevent Ado-mediated inhibition of CD8^+^ TILs, probably by inhibiting ODC and even disrupting spermine synthesis, leading to a significant reduction in total polyamines (265, 266).

### 4.2 Polyamines are central determinants for the fidelity of Th1 cell subsets

T lymphocyte response is necessary for the host to defend against pathogens. According to the difference of antigen and cytokine microenvironment during activation, human CD4^+^ effector T cells can differentiate into at least four main subtypes, including Th1, Th2, Th9, and Th17 (267–269). The main inducers of Th1 cells are IL-12 and IFN-γ. IL-12 is produced by antigen-presenting cells and interacts with its receptors to induce the expression STAT4 and T-bet, the main transcription factor of Th1 cells. T-bet directly binds to the promotor of various Th1 specific genes and promotes their expression (270). T-bet can also negatively regulate the expression of Th2 and Th17 specific genes to inhibit the differentiation of Th2 and Th17 cells. STAT4 can directly bind to the Ifng locus and stimulate IFN-γ production. The cooperation of STAT4 and T-bet will induce the greatest amount of IFN-γ. Therefore, in the absence of STAT4, T-bet alone cannot induce an optimal expression of IFN-γ (270–272).

Metabolic reprogramming is an important factor in the activation and differentiation of T cells (242). Recent studies have shown that polyamine metabolism is a major determinant of fidelity of helper T cell lineages (223). Ornithine decarboxylase is a key enzyme in polyamine synthesis. Lack of ornithine decarboxylase leads to the serious failure of CD4^+^ T cells to adopt the correct subgroup specification, which is highlighted by the ectopic expression of a variety of cytokines and lineage-defining transcription factors across Th cell subsets (223). Even though spermidine does not inhibit the cell proliferation or cytokine production of Th1 cells, T-bet^+^ T cells were slightly reduced when stimulated with higher doses of spermidine, indicating that spermidine may interfere with the Th1 cell differentiation process (273). The expression of inducible co-stimulator (ICOS) is an important indicator of the anti-tumor response of Th1 cells (274, 275), and serves as a new potential biomarker for T cell-mediated immunotherapy response (276–278). However, PD-1 down-regulates ICOS on CD4^+^ T cells, which inhibits the differentiation of CD4^+^ T cells into Th1 cells and affects the anti-tumor response of Th1 cells (256, 279). Polyamines may affect the expression of ICOS in Th1 cells through PD-1 and then regulate the immune function of Th1 cells, while PBT (polyamine Blocking Therapy) has been shown to enhance the anti-tumor effect of PD-1 blockade. These data imply that polyamine and PD-1/PD-L1 may synergistically contribute to impaired functions of effector T cells and then tumor growth (231, 256, 279). Meanwhile, polyamines can also regulate the function of Th1 cells by regulating the production of cytokines. For instance, polyamines, especially spermidine, have been reported to inhibit the production of IL-12 in immune cells, thus resulting in a reduced expression of STAT4 and T-bet, and ultimately, a significant reduction in IFN-γ production (45, 123, 250, 270–272). Taken together, polyamines may play important roles in regulating the antitumor immunity of Th1 cells.

### 4.3 Polyamine-Treg cells: Inhibitory fuel for TIME

Tregs cells are a small subset of CD4^+^ T lymphocytes (about 5%), which are composed of several cell subgroups with similar phenotypes and can inhibit the function of autologous conventional T cells (Tconv) (280, 281). There are two main subgroups of Treg cells: natural Treg cells and adaptive Treg cells. Natural Treg cells originate from the thymus and mediate inhibition through cells contact-dependent mechanism. Adaptive Treg cells, also called type 1 regulatory T cells (Tr1), are induced in the periphery in response to environmental signals, including antigens, IL-2, TGF-β, IL -10 and cAMP (282, 283). The homing of Treg cells is a key step in the initiation and spread of immunosuppressive TME (284). In TIME, Tregs cross-talk with other types of cells, including infiltrating effector T cells, stromal cells, and tumor cells. Treg cells contribute to the immunosuppressive TME through multiple mechanisms, such as inhibiting the maturation of antigen presenting cells (APC), the secretion of pro-inflammatory cytokines and the production of cytotoxic granzymes and perforin by Th1 and CD8^+^ T cells (285). Studies also indicate that Tregs can also support tumor progression through some non-immune mechanisms, such as promoting angiogenesis, proliferation, and metastasis of tumor cells (286–288).

Several lines of evidence have implicated the important role of polyamines in regulating Tregs (289). A recent study has demonstrated that polyamine-related enzyme expression was significantly enhanced in pathogenic Th17 cell but suppressed in Treg cells, while pharmacological and genetic ablation of polyamine metabolism inhibited Th17 cytokine production and reprogrammed the transcriptome and epigenome of Th17 cells toward a Treg-like state as evidenced by enhanced Foxp3 expression (290). Spermidine can also regulate T cell development and enhance the differentiation of mouse and human naive T cells into Treg cells in an autophagy-related manner. The increased synthesis of polyamines in tumor cells may lead to increased secretion of spermidine, which in turn may damage anti-tumor immunity by promoting Treg cells (273). In the process of polyamine catabolism, a large amount of reactive oxygen species (ROS) is produced (77, 79). In TME, ROS can affect the function of immune cells, e. g. the inhibition or activation of Treg functions depending on its concentration (291, 292). In general, ROS at a low level suppresses the function of Treg cells. *In vitro*, neutrophil cytoplasmic factor 1-deficient mice have lower ROS levels than wild-type mice, and the Treg cells isolated from neutrophil cytoplasmic factor 1-deficient mice have weakened functions. In addition, thiol-bearing antioxidants or NADPH oxidase inhibitors reduce ROS levels and then can block or attenuate Treg-mediated inhibition of CD4^+^ effector T cells (293). However, in psoriatic dermatitis, elevated ROS levels can induce hyperfunction of Treg cells (294). Moreover, Treg cells are hyperactive in the culture of 3-dimethoxy-1,4-naphthoquinone (DMNQ), which can induce an increase in ROS levels in a dose-dependent manner (295). It was reported that spermidine ameliorated Dextran Sulfate Sodium -induced inflammatory bowel disease (IBD) in mice by promoting M2 macrophage polarization by inducing mitochondrial reactive oxygen species (mtROS). ROS are key signaling molecules that play a critical role in tumor immunity. Whereas, how ROS production during polyamine catabolism could affect the immune function of Tregs cells, and to what extend would ROS contribute to polyamines’s function in Treg cells regulation, remain to be further investigated.

## 5 Clinical studies of polyamine blockade therapy for cancer

Due to the general elevated level of polyamines in TIME and their wide spectrum effects on tumor and immune cells, polyamine blockade therapy (PBT) is emerging as a novel adjuvant therapy of both chemo- and immune-therapies for a variety of cancers (9, 67, 296). DFMO is a potent, highly specific enzyme-activated, irreversible inhibitor of ODC activity (297–299). DFMO has shown excellent promise in chemoprevention and/or treatment of cancer (9). However, a major disadvantage of DFMO as monotherapy is the compensatory increase in polyamine transport when polyamines are depleted. Therefore, the use of nontoxic polyamine transport inhibitors in combination with DFMO to deplete polyamine levels is a more promising area, which is PBT therapy (300). The most exciting finding is that PBT therapy not only depletes polyamines in tumor cells, but also promotes anti-tumor immune responses, resulting in greater anti-tumor effects than expected. In immunocompetent mouse models of lymphoma, melanoma, and colon cancer, treatment with DFMO in combination with AMXT 1501 inhibited tumor growth by reducing tumor-infiltrating myelosuppressor cells and increasing CD3^+^ T cells (68). In addition to AMXT1501, DFMO can also be used in combination with different polyamine transport inhibitors (Trimer PTIs) to increase granzase B and IFN-γ and activate effector T cells, ultimately inhibiting tumor-promoting microenvironment and increasing antitumor immune responses (67). To date, numerous inhibitors of polyamine metabolism-related enzymes or polyamine transport have been shown to possess potent antitumor effects both *in vitro* and in preclinical cancer models, and several of them have been moved into clinical trials for treating a variety of cancer (**Table 1**).

**Table 1:** Polyamine metabolism interventions in cancers: Clinical trials*.

Inhibitor	Target	Cancer	Status	Phase	Interventions	Plus drugs	Immune cells that may be involved
DFMO	ODC	Prostate Cancer	Completed	2	DFMO in high-risk therapy	–	–
		Prostate Cancer	Completed	2	DFMO to prevent recurrence	Bicalutamide	–
		Non-melanoma Skin Cancer	Recruiting	2	DFMO for chemoprophylaxis	Solaraze	–
		Non-melanomatous Skin Cancer	Completed	3	DFMO to prevent recurrence	–	–
		Non-melanomatous Skin Cancer (Precancerous/ nonmalignant condition)	Completed	2	DFMO to prevent recurrence	Triamcinolone	–
		Bladder Cancer	Completed	3	DFMO to prevent recurrence	–	–
		Cervical Cancer (Precancerous condition)	Completed	2	DFMO to prevent recurrence	–	–
		Esophageal Cancer	Completed	2	DFMO to prevent recurrence	–	–
		Colorectal Cancer (with familial adenomatous polyposis)	Completed	2	DFMO in high-risk therapy	Celecoxib	–
		Colorectal Cancer (Precancerous condition)	Completed	3	DFMO to prevent recurrence	Sulindac	–
		Colorectal Neoplasms	Recruiting	3	DFMO to prevent recurrence	Sulindac	–
		Adenomatous Polyp	Completed	2	DFMO in high-risk therapy	Aspirin	–
		Gastric Cancer	Recruiting	2	DFMO in high-risk therapy	–	–
		Anaplastic Astrocytoma	Recruiting	3	DFMO to prevent recurrence	Lomustine	–
		Medulloblastoma	Recruiting	2	DFMO in high-risk therapy	–	–
		Neuroblastoma	Recruiting	2	DFMO to prevent recurrence	Etoposide	–
		Neuroblastoma	Active, not recruiting	2	DFMO to prevent recurrence	–	–
		Neuroblastoma	Active, not recruiting	2	DFMO to prevent recurrence	–	–
		Neuroblastoma	Active, not recruiting	1	DFMO to prevent recurrence	Celecoxib, Topotecan, Cyclophosphamide	–
		Neuroblastoma	Active, not recruiting	1/2	DFMO to prevent recurrence	Bortezomib	–
		Neuroblastoma	Recruiting	2	DFMO to prevent recurrence	–	–
		Neuroblastoma	Completed	1	DFMO to prevent recurrence	Etoposide	–
		Neuroblastoma	Recruiting	2	DFMO in high-risk therapy	Ceritinib, Dasatinib, Sorafenib, Vorinostat	–
		Neuroblastoma	Suspended (Scheduled Interim Monitoring)	2	DFMO to prevent recurrence	Dinutuximab, Sargramostim, Temozolomide, Irinotecan Hydrochloride	–
BENSpm	SSAT, SMOX	Hepatocellular Carcinoma	Terminated	1/2	BENSpm in high-risk therapy	–	–
PG-11047	ODC, AMD1, SRM, SMS, SSAT, SMOX	Solid Tumors	Completed	1	PG-11047 in advanced refractory therapy	–	–
		Solid tumors and lymphoma	Completed	1	PG-11047 in advanced therapy	Gemcitabine, Docetaxel, Bevacizumab, Erlotinib, Cisplatin, Sunitinib 5-flurouracil/leucovorin	Lymphocytes, Macrophages, NK cells
		Lymphoma	Completed	1	PG-11047 to prevent recurrence	-	Macrophages, Lymphocytes, NK cells
AMXT 1501	Polyamine transport	Solid Tumors	Recruiting	1	AMXT 1501 in advanced therapy	DFMO	–

*All clinical trials on cancers intervention are based on polyamine level inhibition, as listed in the https://clinicaltrials.gov/, query date Mar. 4, 2022.

In addition to clinically tested inhibitors of enzymes involved in polyamine metabolism or polyamine transport, there are a number of newly discovered inhibitors that were not initially used to inhibit polyamine levels. Clofazimine (CLF) is a riminophenazine-based drug approved by the US FDA for the treatment of leprosy and tuberculosis (301, 302). CLF plays A role in tumor xenografts by inhibiting Kv1.3 potassium channels, interfering with Wnt signaling, or enhancing phospholipase A2 activity (303–307). Some of these effects of CLF can be explained by CLF-dependent inhibition of polyamines, as polyamines have previously been shown to inhibit phospholipase A2 and C activities (308). In addition, CLF was found to inhibit multiple myeloma through the Aryl hydrocarbon receptor/polyamine biosynthesis axis (309). The Aryl hydrocarbon receptor (AHR) is a direct transcriptional activator of ODC1 and AZIN1. CLF treatment reduced the binding of AHR to the promoters of AZIN1 and ODC1 in a dose-dependent manner, accompanied by a decrease in the levels of putrescine, spermidine and spermine. Not only this, but CLF can also induce secretion of acetylated polyamines (catalyzed by SSAT) as well as increased protein levels of SMOX, suggesting that CLF promotes polyamine catabolism (309). Therefore, it is not necessary to only use traditional polyamine inhibitors to intervene polyamine metabolism, but also can be combined with other drugs to intervene polyamine metabolism, or combined with other immunotherapy modalities. However, these require further investigation to realize the full potential of this strategy.

## 6 Conclusions

**Figure 3:**
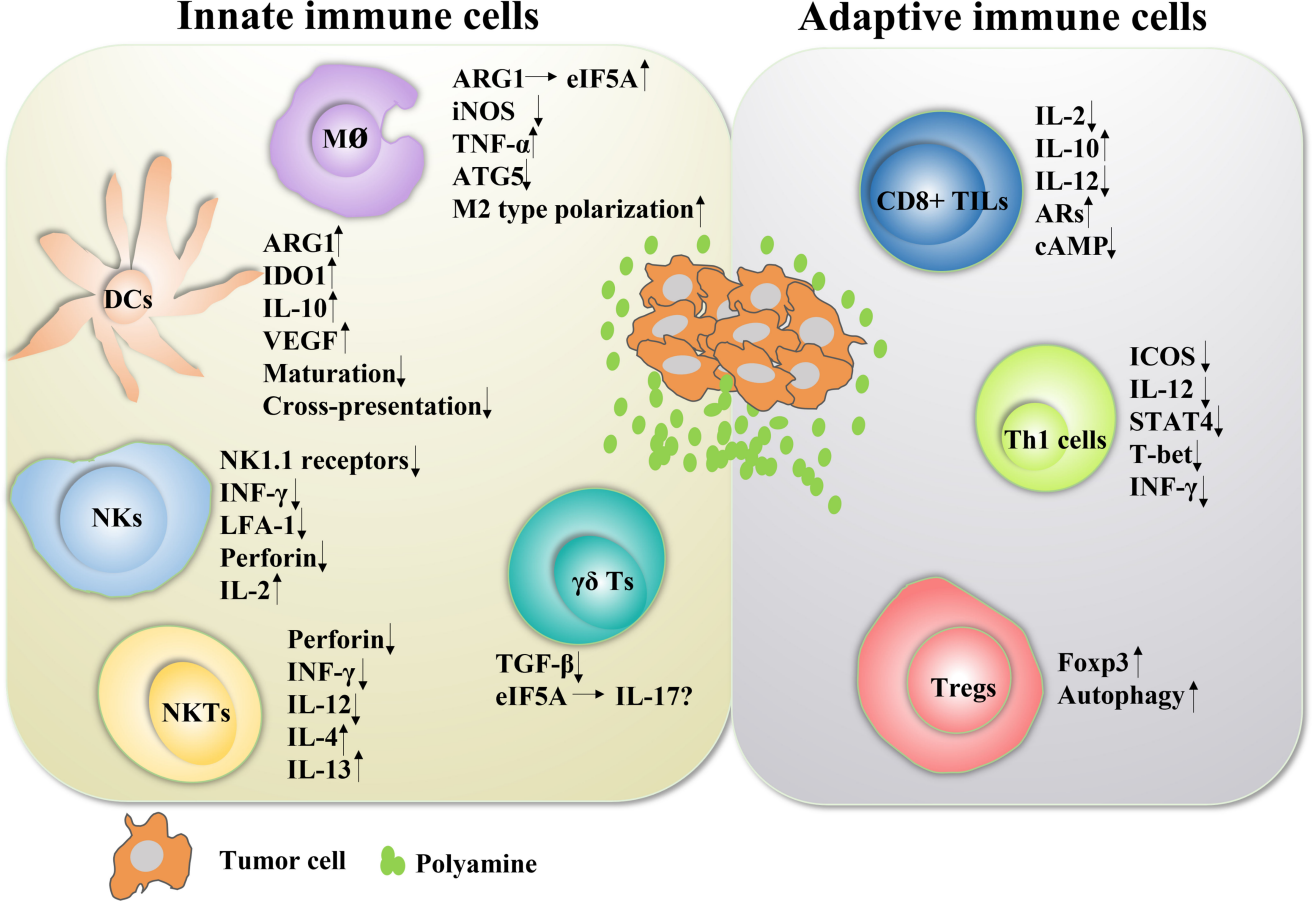
The role of polyamines in both innate and adaptive immune responses in cancer Polyamines and their key enzymes can reshape the tumor immune microenvironment through a variety of transcription factors or cytokines, even have dual roles. The polyamine-eIF5A-hypusine axis regulates macrophage polarization, especially polyamines tend to promote the polarization of M2-type macrophages. Polyamines also negatively regulated the functions of DC cells, NKT cells, CD8+ TILs and Th1 cells, and positively regulated the functions of Treg cells. For NK cells, polyamines are a double-edged sword. In fact, the tumor immune microenvironment is mutually influenced and restricted by a variety of factors. The regulation of polyamines in regulating the function of immune cells is not absolute, which will change according to the changes of tumor immune microenvironment.

Despite extensive research in the field of polyamines and cancer, the role of polyamines in immunomodulatory function in the complex TIME environment remains uncertain, especially the mechanism by which they promote tumor immune evasion. Various inhibitors utilizing polyamine depletion strategies are currently being tested in clinical trials. DFMO, a specific inhibitor of ODC, shows excellent promise in chemoprevention and/or treatment of cancer. Moreover, recent evidence suggests that PBT therapy can mediate the remodeling of the immune landscape of the tumor microenvironment, particularly to promote antitumor immune responses. Emerging evidence in preclinical models of inflammation demonstrates the critical regulatory role of polyamines in immune cell lineage specification, proliferation, and function (**Figure 3**). Furthermore, the combination of polyamine blockade and checkpoint immunotherapy (anti-PD1 or anti-PDL1 immunotherapy) has yielded exciting results in multiple cancer models in mice. All these reports may provide a rationale for utilizing polyamine depletion strategies to promote antitumor immune responses. In fact, the effect of polyamines on immune function was discovered in 1977, and in this pioneering work, exogenous polyamine administration suppressed innate and adaptive immune responses in mouse splenocytes. After decades of intensive research and thousands of studies published, the effects of polyamines on immunity and cancer are surprising. However, the studies on these immune functions are not comprehensive, mainly focusing on macrophages and T lymphocytes, and there are significant differences between different cell types and different diseases. Therefore, it is necessary to further explore the role of polyamines in different tumor immune microenvironments.

## Author contributions

All authors contributed significantly to the drafting and editing of this manuscript. JZ, XQ, YL conceived the manuscript idea and wrote the manuscript. JL, HZ and JZ revised the manuscript content. ML, ZY, and BL created the manuscript tables and figures. All authors contributed to the article and approved the submitted version.

## Funding

This study was supported by grants from the Dongguan Social Science and Technology Development Project (20211800904532), Natural Science Foundation of Guangdong Province (2021A1515012054, 2021B1515140066, 2019A1515110042, 2019A1515011713), Characteristic Innovation Experimental Project of Ordinary Universities in Guangdong Province (2020KTSCX044), Research Foundation of Guangdong Medical University for Ph.D. Staff (GDMUB2020017), the Open Program of Guangdong Provincial Key Laboratory of Medical Molecular Diagnostics (GPKLMMD-OP202107), the Medical Science Foundation of Guangdong Province (A2021438, A2020331, A2020211).

## Conflict of interest

The authors declare that the research was conducted in the absence of any commercial or financial relationships that could be construed as a potential conflict of interest.

## Publisher’s note

All claims expressed in this article are solely those of the authors and do not necessarily represent those of their affiliated organizations, or those of the publisher, the editors and the reviewers. Any product that may be evaluated in this article, or claim that may be made by its manufacturer, is not guaranteed or endorsed by the publisher.
